# 3D Vehicle Trajectory Extraction Using DCNN in an Overlapping Multi-Camera Crossroad Scene

**DOI:** 10.3390/s21237879

**Published:** 2021-11-26

**Authors:** Jinyeong Heo, Yongjin (James) Kwon

**Affiliations:** Department of Industrial Engineering, Ajou University, Suwon 16499, Korea; hjy9061@ajou.ac.kr

**Keywords:** camera calibration, multi-object tracking, overlapping multi-camera crossroad scene, 3D bounding box estimation, 3D trajectory extraction

## Abstract

The 3D vehicle trajectory in complex traffic conditions such as crossroads and heavy traffic is practically very useful in autonomous driving. In order to accurately extract the 3D vehicle trajectory from a perspective camera in a crossroad where the vehicle has an angular range of 360 degrees, problems such as the narrow visual angle in single-camera scene, vehicle occlusion under conditions of low camera perspective, and lack of vehicle physical information must be solved. In this paper, we propose a method for estimating the 3D bounding boxes of vehicles and extracting trajectories using a deep convolutional neural network (DCNN) in an overlapping multi-camera crossroad scene. First, traffic data were collected using overlapping multi-cameras to obtain a wide range of trajectories around the crossroad. Then, 3D bounding boxes of vehicles were estimated and tracked in each single-camera scene through DCNN models (YOLOv4, multi-branch CNN) combined with camera calibration. Using the abovementioned information, the 3D vehicle trajectory could be extracted on the ground plane of the crossroad by calculating results obtained from the overlapping multi-camera with a homography matrix. Finally, in experiments, the errors of extracted trajectories were corrected through a simple linear interpolation and regression, and the accuracy of the proposed method was verified by calculating the difference with ground-truth data. Compared with other previously reported methods, our approach is shown to be more accurate and more practical.

## 1. Introduction

With the development of intelligent transportation systems (ITS), it is possible to obtain a large amount of vehicle trajectory data that reflect the movement of vehicles on the road from fixed cameras [1]. These data can be used to analyze traffic behavior such as speed, lane change, violation of the traffic rules [2], and traffic flow [3]. In addition, it can be used not only for traffic management and control [4] and real-time traffic situation state estimation [5], but also for accident and dangerous situation recognition and prediction [6,7,8,9,10,11]. Hence, the practicality of using vehicle trajectories has become invaluable. Trajectory mainly refers to a series of 2D coordinates of moving objects in 2D space. Currently, with the development of deep convolutional neural networks (DCNNs), 2D trajectory in video images can be obtained using excellent object detection and tracking methods. In addition, it is possible to extract trajectories of multiple objects at the same time (i.e., multi-object tracking) [12,13]. However, compared to 3D trajectories, 2D trajectories do not include any physical information of objects in the real world; thus, so it is difficult to apply them in practical applications such as collision detection and warning [14] and traffic accident situation reconstruction [15] in autonomous driving. Therefore, accurately quantifying 3D vehicle trajectories can be invaluable.

If only an image-based method is used to obtain 3D vehicle trajectory, it should be based on a 2D object detector and camera calibration. Recently, various methods for 3D vehicle trajectory extraction have been studied, according to geometric feature methods and road environments. Seong et al. [16] proposed a method for correcting vehicle trajectory by extracting the center position on the basis of the geometric characteristics of the vehicle moving according to a 2D bounding box for errors caused by lens distortion and low camera perspective in CCTV at intersections. Kocur et al. [17] proposed a method for estimating the 3D bounding box and velocity of vehicle through perspective transformation based on camera calibration and a known vanishing point geometry from a single-camera scene. The six degrees of freedom (DoF) and dimensions of the vehicle are determined according to one vanishing point, known width, and known length (VWL), based on road edge lines. Thus, this method can obtain a 3D bounding box that is robust to changes in orientation. Although methods for obtaining 3D vehicle trajectories from a single camera have been widely and maturely used, trajectory results are not accurate under conditions such as narrow visual angle, low camera perspective, vehicle occlusion, and a lack of vehicle poses and dimensions.

To solve these problems, there are methods to extract 3D vehicle trajectories using multiple cameras with or without overlapping areas. Compared with single-camera methods, these methods can resolve narrow visual angles with vehicle occlusion and obtain continuous 3D trajectory in a whole space. They usually perform two additional essential steps: (1) trajectory reconstruction; (2) multi-camera vehicle matching. Since multiple camera scenes have different viewpoints, trajectories extracted from each single-camera scene must be reconstructed into a whole space, and continuous vehicle trajectories can be obtained through multi-camera vehicle matching and vehicle reidentification.

Peng et al. [18] proposed a method for extracting vehicle trajectories through CNN-based multi-object tracking in a nonoverlapping multi-camera scene and visualizing them on a satellite map through calculation with a homography matrix. In this method, vehicle matching is performed using CNN features to obtain continuous vehicle trajectories, but it does not contain 3D physical information of vehicles. Castaneda et al. [19] proposed a multi-camera detection and vehicle tracking method in nonoverlapping tunnel scenes using optical flow and Kalman filters. This method can be combined with camera-to-camera vehicle travel time and lane position constraints to obtain continuous vehicle trajectory, which can solve vehicle occlusion problems to some extent. However, the physical location of vehicle trajectory in 3D space is still not available. Tang et al. [20] proposed a method for extracting vehicle physical information and 3D trajectories using multiple cameras on a straight road. The proposed algorithm generates a panoramic image to unify the multi-camera scene perspective into one 3D physical space to extract continuous 3D vehicle trajectories. Then, 3D trajectories are extracted by estimating dimensions of vehicles through camera calibration and one VWL-based geometric constraint, calculating projection centroids of 3D boxes, and projecting them onto the panoramic image.

As mentioned in the related methods above, there are various methods for extracting 3D vehicle trajectories. However, most of these methods are limited to simple traffic conditions or straight roads. The reason is that 3D bounding boxes of vehicles are usually estimated using geometric feature methods such as one or two VWLs and road edge lines. Therefore, in complex traffic conditions such as crossroads and heavy traffic, it is difficult to accurately estimate a rotating trajectory or anomalous trajectory that does not match the traffic flow. In this regard, our proposed method can obtain accurate 3D vehicle bounding boxes for all moving directions of vehicles, and it is robust to narrow visual angles and vehicle/obstacle occlusion under condition of low camera perspective. In addition, it is superior to existing methods in that it can extract continuous 3D vehicle trajectories regardless of road environments (highway, crossroads, etc.). A comparison of several 3D vehicle trajectory methods to our proposed method is summarized in Table 1.

In light of the comparative analysis outlined in Table 1, we propose a DCNN-based continuous 3D vehicle trajectory extraction method in complex traffic conditions considering the problems that exist in current multi-camera 3D trajectory methods. We select a heavy-traffic crossroad and generate our custom dataset using overlapping multiple cameras capable of collecting a wide range of data. Then, using trained DCNN models, 3D trajectories for vehicles in all directions are extracted, and the results are optimized through error corrections. By doing so, the shortcomings listed in the previously conducted similar studies can be rectified, and our extracted data can be used for autonomous driving applications. The remainder of this paper is organized as follows: in Section 2, we describe the traffic data collection and the proposed method to extract 3D vehicle trajectories. The evaluation of experimental results and their analysis are presented in Section 3. Lastly, the conclusions and future work are given in Section 4.

## 2. Materials and Methodology

### 2.1. Data Collection from Heavy Traffic Flow

**Figure 1:**
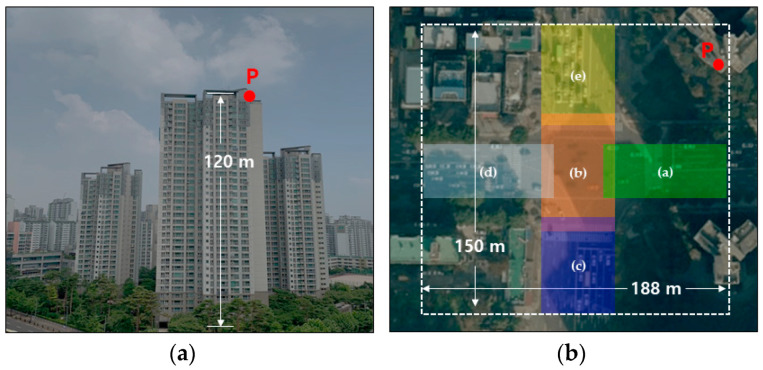
Camera location P at R apartment in South Korea (**a**); orthogonal crossroad map (**b**).

**Figure 2:**
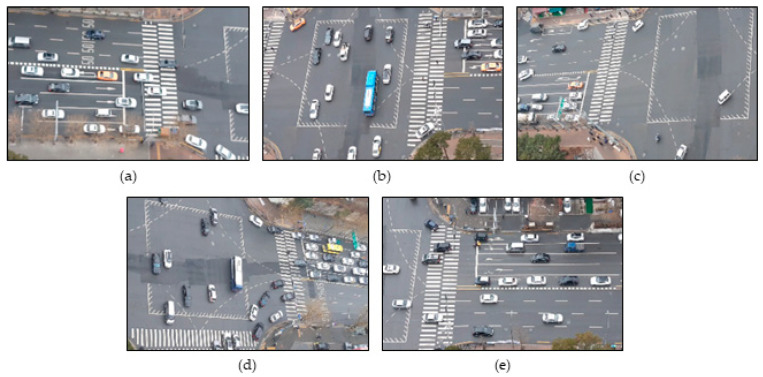
Single-camera scenes from areas (**a**–**e**) in Figure 1b.

We selected a heavy-traffic crossroad in Seoul (the capital of South Korea) to collect a wide range of traffic data in a congested city, and we installed multiple cameras on a high-rise rooftop near the crossroad. The height of the building was about 120 m; thus, it was possible to collect traffic data with a radius of about 75 m from the center of the crossroad. As shown in Figure 1a, five mobile cameras (Samsung Galaxy A2018, Suwon, Korea) were installed in the P position and collected images for about 20 h to obtain a sufficient amount of data. Figure 1b presents an orthogonal crossroad satellite map, showing that all camera images had overlapping areas. Figure 2 shows each single-camera scene taken with five cameras at position P. The specifications of images obtained in this paper were as follows: (1) image resolution: 1920×1080; (2) magnification of camera lens: linear digital zoom × 2; (3) frame speed: 30 fps.

### 2.2. Framework

**Figure 3:**
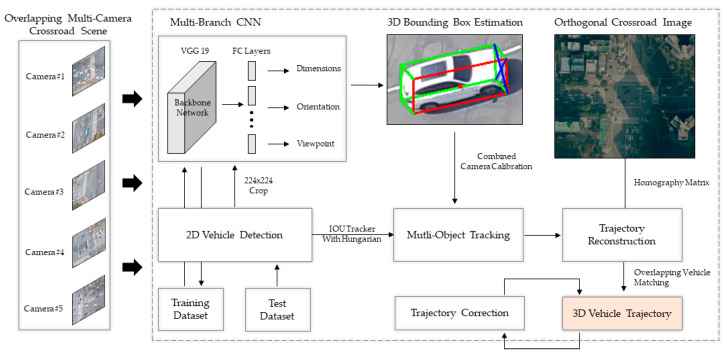
The overall flow chart of the proposed method in this paper.

The overall flow of the proposed method for extracting 3D vehicle trajectories from the overlapping multi-camera scene is shown in Figure 3. First, we generate a dataset for training DCNN models with images obtained in Section 2.1. Then, it detects and tracks vehicles through 2D vehicle detection and multi-object tracking (MOT) algorithms. Second, vehicle physical information (orientation, dimensions, and viewpoint) is inferred from the 2D detection results obtained by a multi-branch CNN model. Then, 3D bounding boxes are estimated by combining the physical information and the camera calibration parameters. Third, trajectories extracted from each single-camera scene with different viewpoints are projected onto the orthogonal crossroad map, which is the 3D ground plane, via calculation with a homography matrix. Finally, 3D vehicle trajectories are extracted by matching vehicles between cameras in the overlapping area. Furthermore, they are optimized through error corrections.

#### 2.2.1. 2D Vehicle Detection and Multi-Object Tracking

To extract vehicle trajectories, vehicle detection and tracking are essential. The 2D detection result contains the center point, width, and height of the 2D bounding box in the image coordinate system, as well as the type of vehicle (car, bus, etc.) and its probability. Currently, with the development of DCNNs, faster and more accurate models have emerged compared to classical object detection methods [21]. Object detection is classified according to the approach: two-stage, e.g., RCNN series [22,23,24], and one-stage, e.g., single-shot multi-box detector (SSD) [25] and ‘you only look once (YOLO)’ series [26,27]. Both methods have their own strengths and weaknesses, and the two-stage method is divided into region proposal and classification stages. This method is not suitable for real-time detection because the detection speed is slow compared to the one-stage method while the accuracy is great. The one-stage method combines both stages into one step. Although the real-time detection performance is excellent, it is relatively inaccurate compared to the two-stage method. In particular, the previous YOLO series did not perform well in detecting small objects and overlapping objects in images. However, recently, YOLOv4 [28] improved both accuracy and detection speed by addressing these problems, and it is widely used in real-time object detection. Therefore, we choose YOLOv4 for 2D vehicle detection in the proposed method.

Trajectories are obtained by tracking the detected vehicle in successive frames. In order to track multiple vehicles simultaneously, multi-object tracking (MOT) algorithm must be applied. Typically, based on SORT (simple online and real time tracking) [12], DeepSort (simple online and real-time tracking with a deep association metric) [13] is one of the most used MOT algorithms, along with tracking using the Kalman filter and IOU (intersection over union) tracker with the Hungarian algorithm [29]. The Kalman filter corrects the error of the tracking result due to missing detection or occlusion, and the IOU tracker connects the detection result of the current frame with the trajectory set of the tracking target. However, as shown in Section 2.1, there is almost no vehicle/obstacle occlusion because it is a high-rise traffic scene. Moreover, when many vehicles are tracked simultaneously, the tracker proposed in [13] using the Kalman filter results in a large amount of computation. Therefore, in order to reduce the computation, we implemented the MOT algorithm using only the IOU tracker without the Kalman filter. IOU denotes the ratio where the two bounding box areas overlap. It is the value obtained by dividing the overlapping area by the union as shown in Equation (1).

(1) $$\mathrm{IOU}\left(\mathrm{Intersection}\;\mathrm{over}\;\mathrm{Union}\right)\;=\;\frac{\mathrm{area}\;\mathrm{of}\;\mathrm{overlap}}{\mathrm{area}\;\mathrm{of}\;\mathrm{union}}.$$

**Figure 4:**
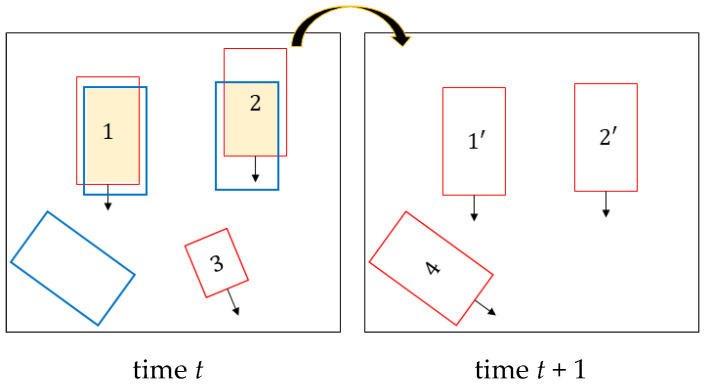
Data association between the detection result and the set of objects being tracked at time *t* in multi-object tracking (blue boxes are objects detected at *t*, red boxes are objects being tracked at time *t* + 1: 1 and 2 were tracked, 3 was deleted, and 4 is a new object).

Using Equation (1), vehicle tracking can be processed by calculating the similarity between detection results of the current frame and the set of trajectories of the tracking target. However, MOT can be affected by several factors that can lead to abnormal tracking, such as missing detection, occlusion, and data association problems. Figure 4 shows an example of an MOT scenario in which these factors appear.

As shown in Figure 4, the red boxes 1, 2, and 3 at time *t* are the objects currently being tracked. Assuming that the blue boxes are new objects detected at time *t*, if IOU values are greater than the user threshold (this value ranges from 0 to 1 and usually performs best when the value is 0.5) between red boxes and blue boxes, the same IDs are assigned, and locations are updated next frame. For example, 1 and 2 have updated locations $1^{\prime }\;\mathrm{and}\;2^{\prime }$ at time t+1, which are positions of the blue boxes at time t. In case 3, box 3 is removed at t+1 due to missing detection. A blue box that does not overlap any of the objects at time t appears at t+1 as a new object assigned ID 4. In this way, the IOU tracker method calculates IOU values between the existing tracked objects and the newly detected objects in successive frames and compares whether they are the same. Then, the ID assignment can be calculated using the Hungarian algorithm. The set of n tracked objects is $T_{n}\;=\;\left\{t_{1},t_{2},\ldots ,t_{n}\right\},\;n\;\in \;N$, and the set of m detected objects in the current frame image $D_{m}\;=\;\left\{d_{1},d_{2},\ldots ,d_{m}\right\},\;m\;\in \;N$; it is possible to solve the matching degree between the tracking objects set $T_{n}$ and the detected objects set $D_{m}$ in the current frame image with the minimum allocation problem. This is a method of finding matching pairs between objects of two sets that are most similar. First, when m and n are given for sets D and T, respectively, the matching matrix, $M_{m\times n}$, is defined as in Equation (2). Here, $iou_{d_{m},t_{n}}$ corresponds to the cost of the IOU value, which is calculated between the object $d_{m}$ and the tracking object $t_{n}$, detected in the current frame image.

(2) $$M_{m\times n}=(\begin{array}{ccccc} iou_{d_{1},t_{1}} & & iou_{d_{1},t_{2}} & \cdots & iou_{d_{1},t_{n}}\\ iou_{d_{2},t_{1}} & & iou_{d_{2},t_{2}} & & iou_{d_{2},t_{n}}\\ & \vdots & & \ddots & \vdots \\ iou_{d_{m},t_{1}} & & iou_{d_{m},t_{2}} & \cdots & iou_{d_{m},t_{n}} \end{array}),\;m,n\;\in \;\left\{1,2,\ldots ,N\right\}.$$

The data association is calculated using the Hungarian algorithm. To change to the minimum assignment problem, the IOU values of $M_{m\times n}$ are multiplied by a negative number.

(3) $$A_{d_{index},t_{index}}\;=\;L\left(-M_{m\times n}\right),\;m,n\;\in \;\left\{1,2,\ldots ,N\right\}.$$

In Equation (3), the function L is a linear assignment function that returns a matrix $A_{d_{index},t_{index}}$. Here, the $A_{d_{index},t_{index}}$ represents the matching relationship between sets D and T as an index matrix. The tracking between the current frame and the next frame can be identified through the matching index pair. However, as shown in Figure 4, box 3 is a case where tracking failed due to missing detection, abnormal tracking, and occlusion. These causes usually depend on the performance of the 2D detector. Finally, the Hungarian algorithm-based IOU tracker can be expressed as an index set, as shown in Equations (4)–(6). Here, $D_{matched}$ is the index set of detected objects included in $A_{d_{index}}$, and $D_{unmatched\_det}$ represents the newly detected ones. The index set of matching objects, $T_{unmatched\_trk}$, is the index set that failed tracking in $T_{n}$.

(4) $$D_{matched}\;=\;\left\{d_{index}|d_{index}\;\in \;T_{n},d_{index}\;\in \;A_{d_{index}}\right\},\;n,\;index\;\in \;\left\{1,2,\ldots ,N\right\}.$$

(5) $$D_{unmatched\_det}\;=\;\left\{d_{index}|d_{index}\;\in \;D_{m},d_{index}\notin A_{d_{index}}\right\},\;m,\;index\;\in \;\left\{1,2,\ldots ,N\right\}.$$

(6) $$T_{unmatched\_trk}\;=\;\left\{t_{index}|t_{index}\;\in \;T_{n},t_{index}\notin A_{t_{index}}\right\},\;n,\;index\;\in \;\left\{1,2,\ldots ,N\right\}.$$

$D_{unmatched\_det}$ is added to set $T_{n}$ to be tracked with newly detected objects. $T_{unmatched\_trk}$ is the set of objects that failed to be tracked due to various causes such as missing detection and occlusion. Since it is important to maintain trajectories through continuous detection in MOT, this problem should be solved by matching the detection result and tracking set, which can be solved by increasing the performance of the 2D object detector or by data association with tight frame intervals.

#### 2.2.2. 3D Bounding Box Estimation Using Multi-Branch CNN

**Figure 5:**
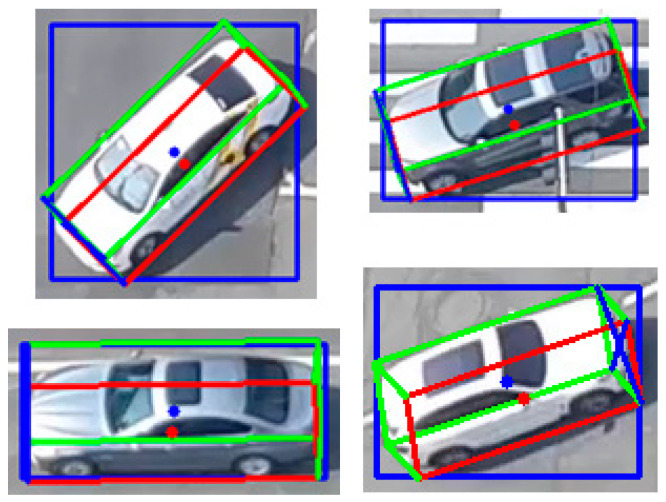
The blue circle is the center of the 2D detection window, and the red circle is the center of the bottom face of the 3D box.

Even though the traffic data were collected from a high-rise, there is still the problem of displacement of the center position of the vehicle due to the camera perspective. The center point of the 2D detection window is different from the actual center of the vehicle, as shown in Figure 5. This issue can be solved by 3D bounding box estimation. To obtain a 3D bounding box, we need to recover the six DoF and dimensions of the vehicle in the real world from the image. Only monocular image-based 3D object detection problems lack a depth information. Usually, 3D properties of a vehicle can be obtained by methods combined with a camera calibration under conditions of one VWL (known width and length), a feature point such as road edge lines, and zero roll and pitch angles of the vehicle. These methods are difficult to apply in complex traffic conditions or in road environments where vehicle movement angles are sensitive, such as crossroads or heavy-traffic conditions. Lingtao et al. [30] proposed a method for regressing orientation, dimensions, and viewpoint from the detection window crop of a vehicle using multi-branch CNN and estimating a 3D bounding box through 2D–3D correspondence constraints in the monocular single camera of the driver’s view. Because this method uses MultiBin estimation for orientation through training, the pose of the vehicle can be obtained regardless of the road environment. Therefore, in this paper, 3D bounding box estimation was performed using the method outlined in [30].

**Figure 6:**
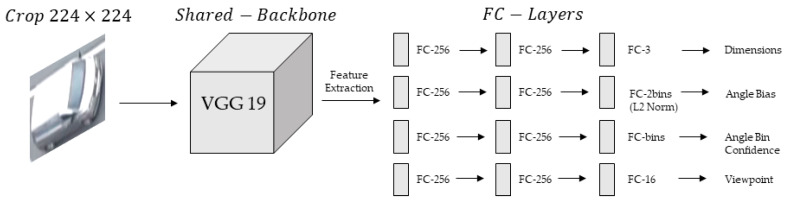
Architecture of the multi-branch network consisting of four branches.

Figure 6 shows the multi-branch CNN architecture. The multi-branch CNN receives the 2D detection window crop from YOLOv4 as an input by resizing it to 224×224. Then, features are extracted from the backbone network, and the orientation, dimensions, and viewpoint of the vehicle are regressed from four branches composed of fully connected layers. Since the VGG 19 was the second-place winner from the 2014 ImageNet challenge and shows good performance, we chose it as the backbone network [31].

**Figure 7:**
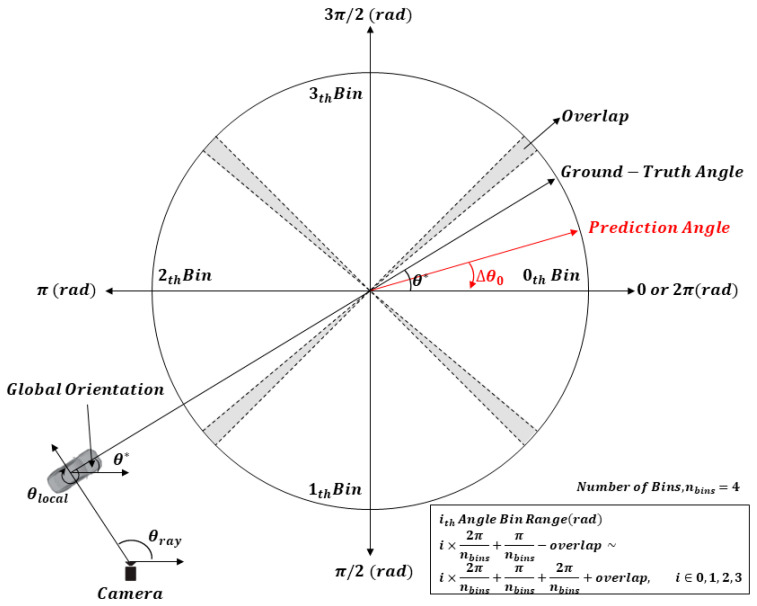
MultiBin structure for orientation estimation (number of bins = 4).

The orientation of the vehicle has an angular range of 360 degrees in a crossroad, and even vehicles with the same global orientation may look different depending on the camera view point. Therefore, it is more effective to estimate the local orientation $\theta_{local}$ from the crop, which is more dependent on the shape of the vehicle, than to directly regress the global orientation. Then, The global orientation $\theta^{*}$ we need can be calculated as $2\pi -\left(\theta_{local}+\theta_{ray}\right)$ using the camera ray $\theta_{ray}$ and the local orientation $\theta_{local}$ of the vehicle in Figure 7. Orientation regression uses the MultiBin (classification of angle bins) method to obtain better angle estimates by decomposing a residual regression between the ground-truth angle and the central angle of the bin [30]. In this paper, four angle bins were used to meticulously estimate the orientation of the vehicle. As shown in Figure 7, the orientation angle is discretized into four overlapping bins to form MultiBin. Each angle bin has a range of $2\pi /n_{bins}$, including the overlap. The angle bin confidence means the probability $c_{i}$ that the prediction angle is contained in the *i*-th bin, and the angle bin confidence loss $L_{conf}$ is trained with a softmax loss. When the prediction angle is the required residual rotation correction $\Delta \theta_{i}$ to the center of that angle bin, the difference from the ground-truth angle is the angle bias. Angle bias is divided into sine and cosine, and an L2 norm layer is added at the end of the branch for angle bias regression. Angle bias loss $L_{ang}$ is defined as cosine similarity, as expressed in Equation (7).

(7) $$L_{ang}\;=\;-\frac{1}{n_{bins}}\sum \cos\left(\theta^{*}-c_{i}-\Delta \theta_{i}\right).$$

MultiBin loss for orientation estimation has two branches (i.e., angle bin confidence and angle bias). As a result, the following parameters are estimated for each bin: $\left(c_{i},\cos\left(\Delta \theta_{i}\right),\sin\left(\Delta \theta_{i}\right)\right)$. The loss $L_{dim}$ for dimensional regression of the vehicle is trained using a simple L2 loss for width, length, and height. The viewpoint is the corresponding configuration in which the 3D bounding box including the vehicle is projected to fit the 2D detection window. Therefore, four out of eight vertices of the 3D box should be projected onto the four edges of the 2D window.

**Figure 8:**
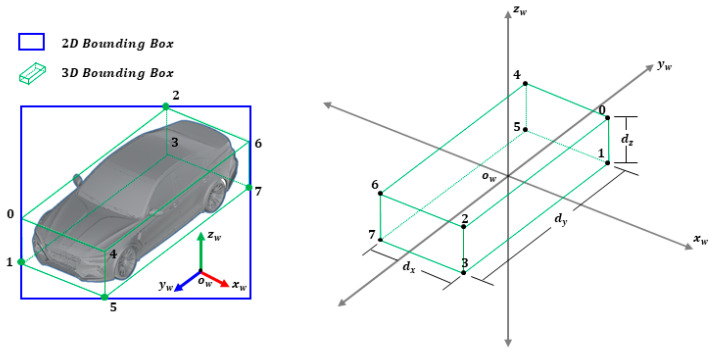
The 3D box viewpoint of the vehicle (four vertices corresponding to four 2D box edges) (**left**) and dimensions of vehicle at original position in the world coordinate system (**right**).

**Figure 9:**
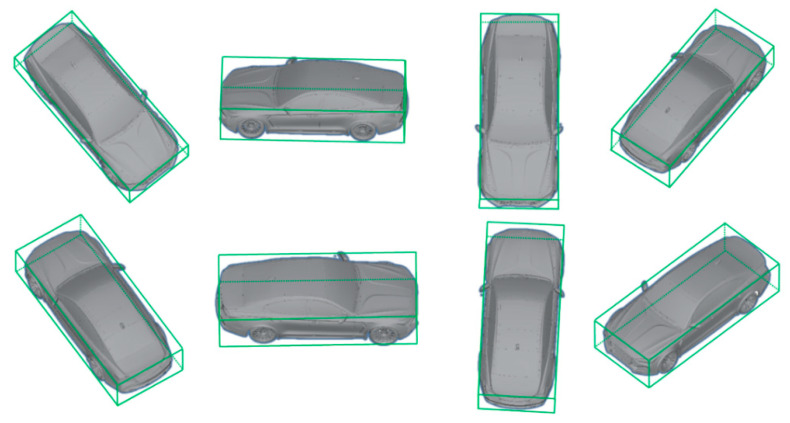
Illustration of eight kinds of observational viewpoints.

For example, as shown in Figure 8, if Arabic numbers are assigned to eight vertices of a vehicle’s 3D box, vertices 1, 7, 2, and 5 are projected to the left, right, upper, and lower edges of the 2D detection window. This is one configuration in which a vehicle can be placed in a 2D window. When the roll and pitch angles of the vehicle on the road are considered as zero, it can be classified into 16 categories according to the observation relationship between the camera and the vehicle. Hence, the viewpoint training of the vehicle is possible through softmax regression. Figure 9 shows some examples in the crossroad according to the observation viewpoint.

Finally, the total loss of the multi-branch CNN is calculated as the sum of the losses of each branch, as shown in Equation (8), where $w_{1},w_{2},w_{3}$, and $w_{4}$ are the weights of each loss.

(8) $$L_{total}\;=\;w_{1}L_{dim}+w_{2}L_{ang}+w_{3}L_{conf}+w_{4}L_{view}.$$

After obtaining the 3D properties of the vehicle through the trained multi-branch CNN model, the next task changes to the problem of determining the location of the 3D box. If dimensions (width, length, height) $D\;=\;{\left[d_{x},d_{y},d_{z}\right]}^{T}$ of the vehicle are known, and the center of the vehicle is the origin, the world coordinates of the vertices of the 3D box (right in Figure 8) can be described as shown in Table 2.

For example, in Figure 8, if the image coordinates of vertices 1, 7, 2, and 5 fitted to the edges of the 2D window are $\left[x_{min},x_{max},y_{min},y_{max}\right]$, then vertex 1 of the 3D box projected to the left edge becomes $X^{0}\;=\;{\left[d_{x}/2,\;d_{y}/2,-d_{z}/2\right]}^{T}$. In this way, given orientation θ and camera intrinsic matrix K, the 2D–3D corresponding constraint for $x_{min}$ can be formulated, as shown in Equation (9).

(9) $$x_{min}\;=\;{\left(K_{3\times 3}\left[R_{3\times 3}^{\theta }|T_{3\times 1}\right]\lceil \begin{array}{c} X_{3\times 1}^{0}\\ 1 \end{array}\rceil \right)}_{x},$$

where ${\left(.\right)}_{x}$ refers to the image coordinate x obtained from perspective projection, $R^{\theta }$ is the rotation matrix parameterized by orientation *θ*, and $T\;=\;{\left[t_{x},t_{y},t_{z}\right]}^{T}$ denotes the transition from camera to the center of the bottom face of the object’s 3D box. Similar equations can be derived for the remaining 2D box side parameters $x_{max},y_{min},y_{max}$. Therefore, by solving T using the four equations, the location of the 3D box can be obtained. This problem can be solved as follows: since there are three unknowns and four equations, it is an overdetermined problem. In order to easily solve this by programming, it can be changed to Equation (10), where I is the identity matrix, and M is a large dummy of parameters. The projection matrix becomes

(10) $$x_{min}\;=\;{\left(K_{3\times 3}\left[\begin{array}{cc} I_{3\times 3} & R_{3\times 3}^{\theta }\times X_{3\times 1}^{0}\\ \;\;\;0 & \;\;\;\;\;\;\;\;1 \end{array}\right]\lceil \begin{array}{c} T_{3\times 1}\\ 1 \end{array}\rceil \right)}_{x}\;=\;{\left(M_{3\times 4}\left[\begin{array}{c} T_{3\times 1}\\ 1 \end{array}\right]\right)}_{x}.$$

In the case of an overdetermined system of linear equations, it is possible to approximate T with the least-squares error estimation equation. Therefore, Equation (10) is expressed as Equation (11) by dividing matrix M into blocks to facilitate matrix calculation.

(11) $$x_{min}\;=\;{\left(\left[\begin{array}{cc} M_{\left[0,0:3\right]} & M_{\left[0,3\right]}\\ M_{\left[1,0:3\right]} & M_{\left[1,3\right]}\\ M_{\left[2,0:3\right]} & M_{\left[2,3\right]} \end{array}\right]\left[\begin{array}{c} T_{3\times 1}\\ 1 \end{array}\right]\right)}_{x}\;=\;{\left[\begin{array}{c} M_{\left[0,0:3\right]}T_{3\times 1}+M_{\left[0,3\right]}\\ M_{\left[1,0:3\right]}T_{3\times 1}+M_{\left[1,3\right]}\\ M_{\left[2,0:3\right]}T_{3\times 1}+M_{\left[2,3\right]} \end{array}\right]}_{x}.$$

If Equation (11) is arranged as a linear expression with respect to T, it can be expressed as Equation (12).

(12) $$\left(M_{\left[0,0:3\right]}-x_{min}M_{\left[2,0:3\right]}\right)T_{3\times 1}\;=\;M_{\left[2,3\right]}x_{min}-M_{\left[0,3\right]}.$$

Since there are four equations, if the left term is rewritten as a 4×3 matrix A and the right term is written as a 4×1 matrix b, it can be expressed as Equation (13).

(13) $$A_{4\times 3}T_{3\times 1}\;=\;b_{4\times 1}.$$

As a result, the linear equation in Equation (14) can obtain the transition T of the 3D bounding box through least-squares fitting by performing the pseudo-inverse matrix of A.

(14) $$T_{3\times 1}\;=\;{\left(A^{T}A\right)}^{-1}A^{T}b.$$

#### 2.2.3. Trajectory Reconstruction and Overlapping Vehicle Matching

**Figure 10:**
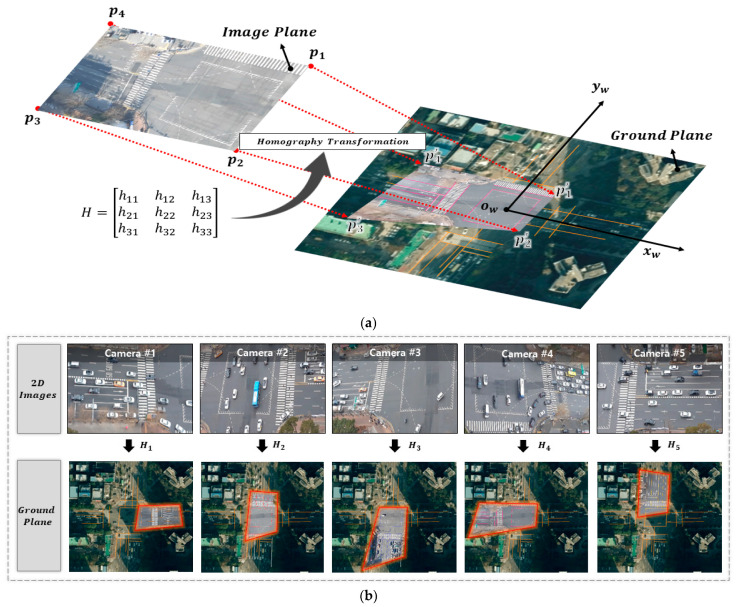
Image projection through homography matrix *H* (**a**); homography transformation between single-camera scenes and ground plane in this paper (**b**).

Although the actual center position of the vehicle is calculated by 3D bounding box estimation in Section 2.2.2, it is not the same space because the reference point is different for each camera. Therefore, in order to complete continuous 3D vehicle trajectory extraction in an overlapping multi-camera scene with different viewpoints, trajectories extracted in 2D images must be reconstructed into a whole space. As shown in Figure 10a, if the orthogonal crossroad map is a ground plane, a certain transformation relationship is established according to the corresponding points projected between the camera image plane and the ground plane. A matrix representing the transformation relationship can be obtained using the homography transformation. The homography transformation only works for planar objects. Hence, we take advantage of the fact that the bottom face of the 3D bounding box is directly above the road. Therefore, trajectories in Section 2.2.2 are calculated using the homography matrix without image transformation. This can now easily be projected onto the ground plane.

In general, homography is expressed as a 3×3 matrix H, and at least four corresponding pairs are required to determine it. That is, in Figure 10a, if points $p_{i}\left(x_{i},y_{i}\right),\;i\;=\;1,2,3,4$ on the image plane are projected as points $p_{i}^{\prime }\left(x_{i}^{\prime },y_{i}^{\prime }\right),\;i\;=\;1,2,3,4$ on the ground plane, respectively, these corresponding points can be expressed as Equation (15).

(15) $$s\left[\begin{array}{c} x_{i}^{\prime }\\ y_{i}^{\prime }\\ 1 \end{array}\right]\;=\;H_{3\times 3}\left[\begin{array}{c} x_{i}\\ y_{i}\\ 1 \end{array}\right]\;i\;=\;1,2,3,4.$$

Matrix H can be applied to any point on the image plane, which can be simply obtained. In addition, the point projected on the ground plane can be converted into the world coordinate system units through the scale factor s. As shown in Figure 10b, all matrices $H_{1},H_{2},H_{3},H_{4},H_{5}$ between the five camera images in Section 2.1 and the ground plane can be obtained. Then, vehicle trajectories of each single-camera scene obtained in Section 2.2.2 are projected onto the ground plane by calculating the homography matrix. However, an overlapping area is generated in the center of the crossroad, and overlapping vehicle matching is required to obtain a unique vehicle trajectory.

For example, in Figure 10b, for any vehicle moving straight from camera 5 to camera 2, two vehicle trajectories coexist in the same position in the overlapping area without vehicle matching. This problem can be solved by detected time interval (i.e., age) and the distance between center points of the vehicles. The detected time interval of the vehicle is based on how much time has elapsed. For a vehicle $v_{i}$ in set V of all vehicles in the overlapping area of the crossroad, if the distance between the center of the *i*-th vehicle is less than the threshold (e.g., 0.5 or 1.0 m), then it is considered the same vehicle. The vehicle ID depends on the older detected time interval, and the remaining IDs are removed. Finally, continuous vehicle trajectory including physical information can be obtained on the orthogonal crossroad map. The overlapping vehicle matching algorithm is described in detail in Algorithm 1.
**Algorithm 1.** Overlapping vehicle matching on the ground plane **Input:**
$V\;=\;\{v_{1},v_{2},\ldots ,v_{n}\}$*, set of all trackers in overlapping area.*** Input:***d, minimum distance to determine if it is the same object.*
 **Output:**
*O, set of trackers remaining after removing duplicate ID in overlapping area.* 
 *//v is a custom class and contains ID, camera type, and age attributes.*
 //id is tracking index of vehicle.
 //$camera\;type\;is\;the\;recording\;area\;\left(center,\;east,\;west,\;south,\;north\right)$. //age is the number of times detected.  **if**
n>0
**then**  **for all**
$v_{i}\;\in \;V$
**do**     $S\leftarrow Set\;of\;those\;less\;than\;the\;distance\;d\;between\;v_{i}\;and\;V_{except\;i}$  **if** S is null then continue  **if**
$camera\;type\;of\;v_{i}\;=\;center$ **then**  //*Center is the intersection.*  **continue**  **else**  *ID*
**of** $v_{i}\leftarrow ID\;of\;\underset{age}{\max}\left(S\right)$  **for each**
*s*
**in**
*S*                  $ID\;of\;S\leftarrow ID\;of\;v_{i}$  $O\leftarrow \mathrm{remove}\mathrm{Duplicate}\mathrm{ID}\left(V,\;\;key\;=\;ID\right)$   //*Remove trackers with duplicate ID*

#### 2.2.4. Vehicle Trajectory Correction

**Figure 11:**
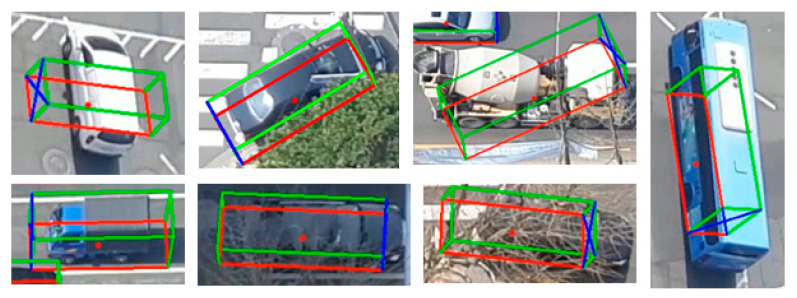
Examples of inaccurate vehicle 3D box estimates based on DCNN models.

In order to obtain a high-accuracy 3D vehicle trajectory, the performance of DCNN-based models is very important. However, it can lead to inaccurate results due to various reasons such as overfitting, underfitting, incorrect labeling, lack of training data, and vehicle/obstacle occlusion. As shown in Figure 11, inaccurate results such as vehicle position, orientation and dimensions have a significant impact on trajectory extraction. The accumulated errors due to these can result in 3D vehicle trajectories with outliers and noise.

We use sampling-based linear interpolation and regression methods to remove outliers and noise from vehicle trajectories extracted by the proposed method. Since any excessive corrections can make the error of the original trajectory data larger, we apply the correction considering the dynamics of the moving object at every interval. In particular, in high-traffic data, the movement of vehicles is slow and stable compared to highways. For example, it is extremely difficult to change the orientation angle of a vehicle by more than 30 degrees or to move more than 10 m within 0.1 s on a crossroad. Taking these general characteristics into consideration, a simple vehicle trajectory correction is possible. The trajectory correction method is described in Algorithm 2. The proposed algorithm first calculates the displacement and angular displacement between times t−1 and t, and, if the threshold α and β are exceeded, it is regarded as an outlier. Then, the values at time t are predicted through previous linear and angular velocities. After that, linear regression equations are obtained using data from both endpoints for each sampling interval γ of the trajectory. Afterward, the noise and vibration are minimized by regressing trajectory data through these linear equations.
**Algorithm 2.** Trajectory correction using linear regression and interpolation **Input:**
$T\;=\;\{t_{1},t_{2},\ldots ,t_{n}\}$*, set of all trajectories of vehicles included position, orientation.* **Input:**
*σ, orientation threshold at which the vehicle can turn for time* Δ*t.* **Input:**
*β, position threshold at which the vehicle can move for time* Δ*t.* **Input:**
*γ, interval frames to interpolate.* **for all** $t_{i}\;\in \;T$
**do** $X\leftarrow \mathrm{Position}\_X\left(t_{i}\right)$//*Set of position trajectories x of*
$t_{i}$   $Y\leftarrow \mathrm{Position}\_Y\left(t_{i}\right)$//*Set of position trajectories y of*
$t_{i}$   $O\leftarrow \mathrm{Orientation}\left(t_{i}\right)$//*Set of orientation trajectories of*
$t_{i}$     **for all** $x_{i}\;\in \;X,y_{i}\;\in \;Y,o_{i}\;\in \;O$ **do**//*Remove outliers*
   difference_o← $\left|o_{i}-o_{i-1}\right|$   difference_d← $\mathrm{Distance}\left(\left|x_{i}-x_{i-1}\right|,\;\left|y_{i}-y_{i-1}\right|\right)$    **if** difference_o>σ
**then**
               $angular\_vel\leftarrow \left(o_{i-1}-o_{i-k}\right)/time\;i-k\;to\;i-1\;$                    $o_{i}\leftarrow o_{i-1}+angular\_vel*\Delta t\;$    **if** difference_d>β
**then**                  $x\_vel\leftarrow \left(x_{i-1}-x_{i-k}\right)/time\;i-k\;to\;i-1$                 $y\_vel\leftarrow \left(y_{i-1}-y_{i-k}\right)/time\;i-k\;to\;i-1$                      $x_{i}\leftarrow x_{i-1}+x\_vel*\Delta t$                     $y_{i}\leftarrow y_{i-1}+y\_vel*\Delta t$   **for**
*interval*
γ $x_{i}\;\in \;X,y_{i}\;\in \;Y,o_{i}\;\in \;O$ **do**//*Interpolation through Linear Regression*   *Calculate the linear equation for*
x,y,o
*on the interval*
i *to*
i+γ **for**
i *to* i+γ **about** x,y,o               $o_{i:i+\gamma }\leftarrow \mathrm{Time}-\mathrm{Orientation}\;\mathrm{Equation}\left(time_{i:i+\gamma }\right)$         $x_{i:i+\gamma }\leftarrow X-Y\;\mathrm{Equation}\left(y_{i:i+\gamma }\right)$ depends on the orientation or *Y*–*X*
                     i←i+γ


## 3. Experiments and Results

We used AMD (Santa Clara, CA, USA) Ryzen 5 3600 6-Core Processor, NVIDIA (Santa Clara, CA, USA) GeForce RTX 2080Ti, 32 GB RAM, and Window 10 operating system for our experiments. Experiments first generated a dataset to train the two neural networks (YOLOv4, multi-branch CNN) mentioned in Section 2.2.1 and Section 2.2.2 on the basis of high-traffic data collected in Section 2.1. Then, 3D vehicle trajectories were extracted on the ground plane (an orthogonal crossroad map) according to the proposed method using the trained network models. The performance of the proposed method was evaluated by calculating the difference between extracted 3D vehicle trajectory results and ground-truth data. Additionally, DCNN-based trained models have a significant impact on vehicle detection and tracking. Thus, vehicle trajectories inevitably contain errors. We tried to minimize the outliers and noise of extracted trajectories using the algorithm proposed in Section 2.2.4 for generated errors. Then, the results before and after the error correction were evaluated. Finally, the effectiveness of the proposed method was verified through result analysis. This section can be divided into two aspects: (1) generation and training of custom dataset to train neural network models necessary for 3D bounding box estimation; (2) 3D vehicle trajectory extraction and result analysis.

### 3.1. Dataset Labeling and Training Results

In this proposed method, since there is no public dataset, we generated our labeled data necessary for training. We developed our customized 3D labeling tool and generated a training dataset. To train the multi-branch CNN, actual measurement information (dimensions, orientation, etc.) of vehicles included in an autonomous driving open-source dataset such as KITTI [32] and BrnoCompSpeed [33] was required. Since it is very difficult to collect a large amount of measured data through sensors, the physical information of vehicles was approximated using the camera intrinsic parameters in the 3D labeling tool, and these were used as ground-truth data. Labeled data included information such as type, coordinates of 2D bounding box, orientation, and actual dimensions (width, length, height) of vehicle. A total of 33,036 data (bike: 3775, car: 23,479, bus: 1772, truck: 3524, Remicon: 486) were generated for the five camera images used in this paper.

**Figure 12:**
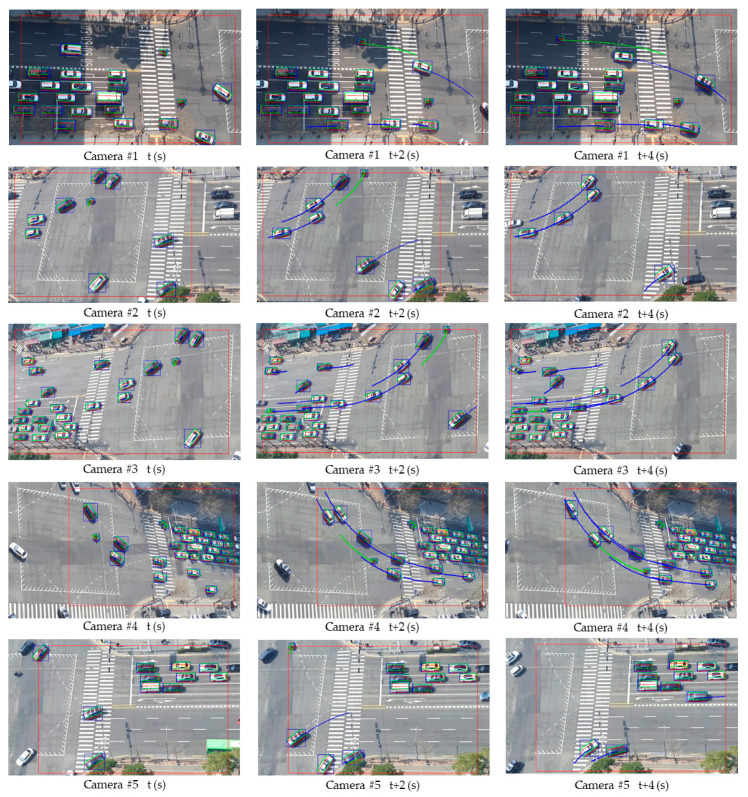
Examples of 2D–3D detection and tracking through models trained in this paper for five cameras (only detected when entering red boxes).

YOLOv4 was trained using the open-source framework Darknet. The multi-branch CNN was trained in the deep learning framework Pytorch environment, and the weighting factors in Equation (8) were set as $w_{1:4}\;=\;\left[4,\;8,\;10,\;8\right]$. Figure 12 shows examples of 2D–3D detection and tracking through these models trained in this paper for five single-camera scenes. It can be seen that the 3D bounding boxes were well estimated for vehicles whose orientation angles were sensitively changed regardless of camera viewpoints.

### 3.2. 3D Vehicle Trajectory Extraction and Result Analysis

**Figure 13:**
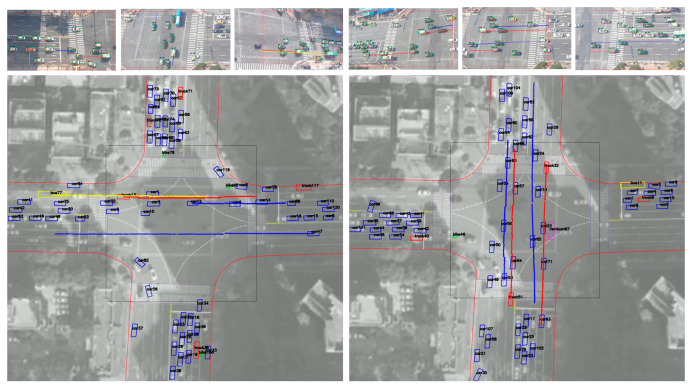
Examples of vehicle trajectories: driving straight (left and right) (**left**); driving straight (up and down) (**right**).

**Figure 14:**
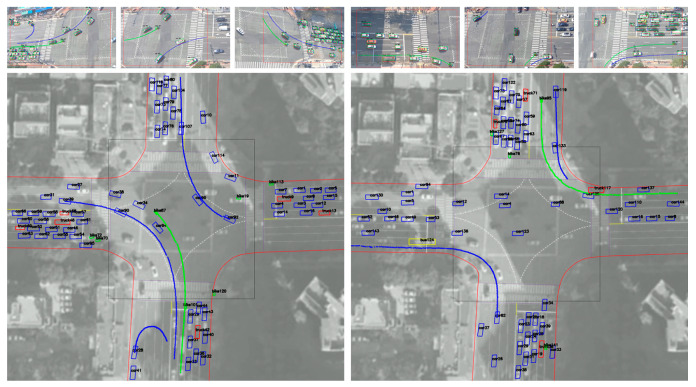
Examples of vehicle trajectories: left turn and U turn (**left**); right turn (**right**).

Figure 13 and Figure 14 show examples of extracted vehicle trajectories from four cases according to the proposed method: (1) straight (left and right); (2) straight (up and down); (3) left turn and U turn; (4) right turn.

**Figure 15:**
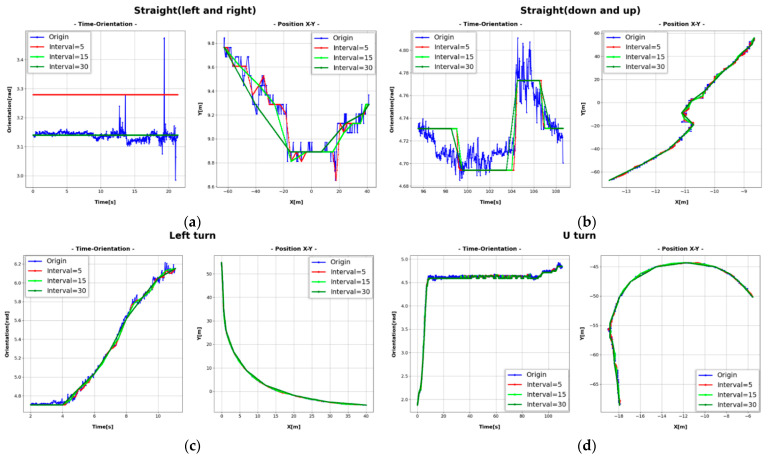
Results for error correction according to intervals of five, 15, and 30 frames with origin trajectory: (**a**) straight (left and right); (**b**) straight (down and up); (**c**) left turn; (**d**) U turn.

As shown in Figure 13 and Figure 14, the color of the vehicle (bike: green, car: blue, bus: yellow, truck: red, concrete mixer: purple) is displayed differently depending on the type. According to the proposed method, trajectories plotted on the orthogonal crossroad map show the path and orientation similar to the movement of real vehicles in camera images. However, when some trajectories were enlarged along the axis, it can be seen that they were not smooth and contained noise and outliers due to cases shown in Figure 11 and inaccurate inference of detectors. Therefore, we corrected errors of the extracted trajectory (time, orientation, and position *x*–*y*) using the algorithm proposed in Section 2.2.4. We selected several examples for different types of driving and corrected the trajectories according to sampling intervals of five, 15, and 30 frames. Then, we compared them on the graph with original trajectories in Figure 15.

Finally, for quantitative evaluation of this proposed method, we calculated the difference between 5612 ground-truth data and the derived samples. The accuracy of dimensions (width, length, height) was very high at 97.2%, 96.1%, and 96.4% using the multi-branch CNN. In high-traffic data, the dimensions showed slight differences for each type of vehicle, which were very robust. Therefore, we evaluated the orientation and position of 3D vehicle trajectories. Orientation was calculated as the average orientation similarity (AOS) [32], and position was calculated for the *x*-axis and *y*-axis as the root-mean-square error (RMSE), defined in Equations (16) and (17).

(16) $$AOS\;=\;\frac{1}{N}\underset{i\;\in \;N}{\sum }\frac{1+\cos\left|\theta_{predicted}^{\left(i\right)}-\theta_{ground-truth}^{\left(i\right)}\right|}{2}\times 100\%,\;i\;=\;1,2,\ldots ,n.$$

(17) $$RMSE\;=\;\frac{1}{N}\underset{i\;\in \;N}{\sum }\|p_{predicted}^{\left(i\right)}-p_{ground-truth}^{\left(i\right)}\|{}_{2},\;i\;=\;1,2,\ldots ,n.$$

As shown in Table 3, even though the origin had the lowest accuracy (79.6%), the remaining data, such as the accuracy of vehicle position (*x* and *y*), reached the centimeter level. In addition, trajectory results corrected according to intervals of five, 15, and 30 frames were very close to ground-truth data. In particular, it is shown that trajectory results with error correction applied every 15 frames (0.5 s) manifested excellent performance. Furthermore, the accuracy of orientation and position was greatly improved compared to the origin. Therefore, the method proposed in this paper can effectively extract 3D vehicle trajectories of vehicles, and its accuracy is further improved through the error correction technique.

## 4. Conclusions

In this paper, we proposed a method to extract 3D vehicle trajectories using deep convolutional neural networks (DCNNs) in an overlapping multi-camera crossroad scene. The main contributions of this paper are summarized as follows:

- A method for estimating 3D bounding boxes of vehicles through YOLOv4, MOT, and multi-branch CNN is proposed on the basis of camera calibration and correspondence constraints.
- A method of processing trajectory reconstruction and vehicle matching to obtain a continuous 3D vehicle trajectory in a multi-camera crossroad scene is proposed.

Compared to existing 3D vehicle trajectory extraction methods, continuous 3D trajectories can be obtained including the vehicle’s physical information, which is helpful for applications in large-scale road scenes. In particular, the proposed method is robust to vehicle occlusion and narrow visual angles. It is also superior to the existing methods in that it is possible to extract 3D vehicle trajectories regardless of the road environment. In the future, it is necessary to develop 2D–3D detectors as a single end-to-end neural network. By doing so, the computation time will be drastically reduced, and real-time processing will become possible. This can be utilized in the V2I (vehicle-to-infrastructure) field. In addition, it should be possible to accurately extract vehicle trajectories not only during the day, but also in various climatic environments such as at night or in rainy weather. Such research is obviously difficult, but it will be of great help to the improvement of autonomous driving technology in the future.

## Figures and Tables

**Table 1:** Comparison of different 3D vehicle trajectory extraction algorithms.

Method	Vehicle Orientation	Vehicle Position	Continuous 3D Trajectory	Multi-Camera Scene	Straight Road	Crossroad
Seong et al. [16]	X	O	O	X	O	O
Kocur et al. [17]	O	O	O	X	O	X
Peng et al. [18]	X	O	X	X	O	O
Castaneda et al. [19]	X	X	X	O	O	X
Tang et al. [20]	O	O	O	O	O	X
Our method	O	O	O	O	O	O

**Table 2:** World coordinates of eight vertices when positioned at the vehicle’s origin.

Number	World Coordinate
0	($d_{x}/2,\;\;d_{y}/2,\;\;d_{z}/2)$
1	($d_{x}/2,\;\;d_{y}/2,\;-d_{z}/2)$
2	($d_{x}/2,\;-d_{y}/2,\;\;d_{z}/2)$
3	($d_{x}/2,\;-d_{y}/2,\;-d_{z}/2)$
4	($-d_{x}/2,\;\;d_{y}/2,\;\;d_{z}/2)$
5	($-d_{x}/2,\;\;d_{y}/2,\;-d_{z}/2)$
6	($-d_{x}/2,\;-d_{y}/2,\;\;d_{z}/2)$
7	($-d_{x}/2,\;-d_{y}/2,\;-d_{z}/2)$

**Table 3:** Quantitative difference between ground-truth data and 3D vehicle trajectory extracted using proposed method.

Our Method	AOS	RMSE (*x*)	RMSE (*y*)
Origin	79.6% (±54°)	29.1 cm	26.5 cm
Interval = 5 frames	99.2% (±10°)	19.5 cm	23.1 cm
Interval = 15 frames	99.8% (±5°)	12.9 cm	15.2 cm
Interval = 30 frames	98.5% (±12°)	26.7 cm	30.5 cm

## Data Availability

The data presented in this study can be available upon request to the corresponding author.
